# Mothers impose physical activity restrictions on their asthmatic children and adolescents: an analytical cross-sectional study

**DOI:** 10.1186/1471-2458-14-287

**Published:** 2014-03-28

**Authors:** Fabianne MNA Dantas, Marco AV Correia, Almerinda R Silva, Décio M Peixoto, Emanuel SC Sarinho, José A Rizzo

**Affiliations:** 1 Hospital Agamenon Magalhães, Pos-graduação em Ciências da Saúde. Universidade Federal de Pernambuco, Recife, Brazil; 2 Universidade de Pernambuco, Petrolina, Brazil; 3 Center for Research in Allergy and Clinical Immunology. Pediatrics Department, Universidade Federal de Pernambuco, Recife, Brazil; 4 Center for Research in Allergy and Clinical Immunology. Clinical Medicine Department - Pneumology, Universidade Federal de Pernambuco, Recife, Brazil

**Keywords:** Asthma, Child, Adolescent, Physical activity, Restriction

## Abstract

**Background:**

Physical activities are important for children and adolescents, especially asthmatics. A significant proportion is considered less active than their non-asthmatic peers and mother’s beliefs about asthma are thought to be a determinant factor.

The research objectives were to investigate whether mothers try to impose limitations on the physical activity (PA) of their asthmatic children/adolescents; identify associated factors; and explore if this attitude has any impact on children’s PA levels.

**Methods:**

In this cross sectional investigation, we studied 115 asthmatics aged between 9 and 19 years and their mothers. Asthma severity, PA level and exercise induced bronchospasm (EIB) were evaluated. Mothers were questioned on their beliefs about physical activity in non-asthmatic and asthmatic children, if they imposed restrictions on their children’s physical activity, on EIB perception and personal levels of anxiety and depression.

**Results:**

Ninety six percent of the mothers answered that PA are important for children and adolescents. Despite this, 37% of them admitted imposing restrictions to their children’s PA. This attitude was associated with mother’s negative opinions about asthmatics doing PA, perception of children’s dyspnea after running on a treadmill, mother’s anxiety level and children’s asthma severity. The mother’s restrictive attitudes were not associated with children’s lower PA levels.

**Conclusion:**

A high proportion of the mothers said that they restrained their asthmatic children from engaging in physical activity. This fact should be recognized by health professionals and discussed with parents and caregivers as these negative beliefs may lead to conflicts and prejudiced attitudes that could discourage children’s involvement in physical activities and sports.

## Background

Regular physical activity (PA) by children and adolescents with asthma is associated both with an improvement of disease control and quality of life and with a reduction in exacerbations, hospital admissions, school absenteeism, unscheduled medical visits and the number of medications used to control asthma [1-5]. Participating in games, playing around and engaging in sports or physical education at school are also activities that promote children’s social inclusion and relationships, thus preventing psychological and social isolation and improving self-esteem, encouraging an active and healthy lifestyle [6].

Physical activities and sports are considered safe for asthmatics, recommended as a part of their treatment and should not be avoided. This is true even in patients with a diagnosis of exercise-induced bronchospasm (EIB) provided they are adequately monitored and prophylactically treated [7-9]. However, there are evidences that children and adolescents with asthma are less active than their non-asthmatic peers and a maternal negative influence has been identified as a limiting factor [10-12]. On the other hand, when encouraged by their mothers, children become more active [13].

As it is usually the mothers who take care of asthmatic children and adolescents, it is possible that those who think that asthma symptoms can be triggered or worsened by taking part in PA do not encourage or even forbid their children from doing so in order to avoid symptoms, contributing to the inactivity of their children [12,14,15]. Other factors may contribute to the mother’s restrictive attitudes such as negative expectations with regard to PA, anxiety, depression, negative experiences, misperceptions, distorted opinions about physical exercise in asthmatics and the lack of adequate information about the disease [10,15-20].

The objective of this study was to investigate whether the mothers in an urban community imposed restrictions on the physical activity of their asthmatic children, to identify possible factors related to this attitude and to check if there was an association between this behavior and the amount of children’s and adolescent’s PA.

## Methods

### Study design and sample

This was an analytical cross-sectional study conducted at the Pulmonary Functional Laboratory of the Hospital das Clínicas at the Federal University of Pernambuco (UFPE) - Brazil. Data were collected between November 2008 and September 2009. The study was approved by the institutional ethics committee and the mothers agreed and signed an informed consent document.

The mothers and their respective offspring aged between nine and nineteen were recruited at a specialized public clinic. The children had been previously diagnosed as having asthma by an allergist or pulmonologist and were referred to the Pulmonary Function Laboratory to investigate EIB.

The patients involved in the analysis did not have an acute exacerbation during the period of data collection, were without symptoms of respiratory infection in the last six weeks and had a forced expiratory volume in one second (FEV_1_) at baseline ≥ 60% of the predicted value. Patients were excluded who did not meet these requirements, were outside the age range, had other conditions that would preclude running on a treadmill, were using oral steroids. Patients unable to perform the forced expiratory maneuver for the spirometry test and those who were accompanied by guardians other than their mothers were also excluded.

Asthma severity was classified according the Global Initiative for Asthma (GINA) criteria as intermittent or persistent, mild, moderate or severe [8]. For the analysis, the first two and the last two categories were grouped. Asthma medications were interrupted according the recommendations of the American Thoracic Society (ATS) for exercise challenge testing [21]. Nutritional status was determined according the criteria of the World Health Organization (WHO) [22].

### Data collection - questionnaires

General data was collected about the patients (age, sex, weight, height and BMI) as well as information concerning family income and the mother’s level of education. To assess the level of physical activity, children and adolescents answered the short version of the International Physical Activity Questionnaire (IPAQ), translated into Portuguese and validated for Brazil [23]. This questionnaire takes into account activities engaged in for at least ten minutes continuously in the previous week based upon reports of exercise frequency, intensity and duration and classifies individuals as being very active, active, irregularly active A and B and sedentary. For the analysis individuals were considered active when they were classified as very active or active and as inactive when they were classified as irregularly active or sedentary [24].

On the same day, mothers were asked to answer a questionnaire to examine if they imposed restrictions on PA as well as their previous perception about children having EIB and their opinions on asthmatics doing PA. PA restrictions imposed by the mothers were determined by the response to the question “Do you prevent your child from taking part in sports or games because of asthma in the period when he/she is not having an attack?”.

The mother’s perception of EIB was evaluated at two points in time: before and after the exercise test. Before the test, the mother’s response to the following question was considered: “Does your child have wheezing or asthma when at play or taking part in sports?” Then, the mother’s perception of EIB was evaluated by requesting them to mark a point on the visual analogue scale (VAS) that consisted of a 10 cm horizontal line, anchored on the far left by the phrase “no shortness of breath,” and on the far right end by “great shortness of breath”. The measurement was made five minutes after children had been running on the treadmill, immediately before the second spirometry test. The point marked was measured in centimeters (1–10 cm) [16].

The mother’s opinions on children in general and children with asthma engaging on physical exercise were evaluated using the following questions adapted from Lang et al. [15]: “Is doing exercise important for children?”, “Are you afraid that your child will fall ill if he/she does exercise?” and “Is exercise dangerous for children with asthma?”. In addition, they also answered the Hospital Anxiety and Depression Scale (HADS) questionnaire - translated into Portuguese and validated for Brazil [25,26].

### Exercise testing

After responding to the IPAQ, patients underwent basal FEV_1_ measurement and then ran on a treadmill (Athletic Way Advanced - Brazil) for 8 to 9 minutes achieving at least 80% of maximum heart rate for the last 6 minutes [21]. FEV_1_ was again measured at 5, 15 and 30 minutes after running. The MicroQuark® spirometer (Cosmed – Rome - Italy) calibrated daily was used for spirometries that were performed according to ATS/ERS recomendations [27]. The test was considered positive for EIB if there was a FEV_1_ reduction ≥ 10% from baseline. The values predicted for FEV_1_ were calculated from the reference equation for Brazilian children and adolescents [28].

All tests were carried out in the morning, in an air-conditioned room (temperature 21°C - 23°C and relative humidity 60% - 80%). Peripheral oxygen saturation and heart rate were monitored continuously using the pulse oximeter (GE® Marquette Solar 8000 - USA) and a heart rate sensor (Polar RS100 - Finland).

**Figure 1:**
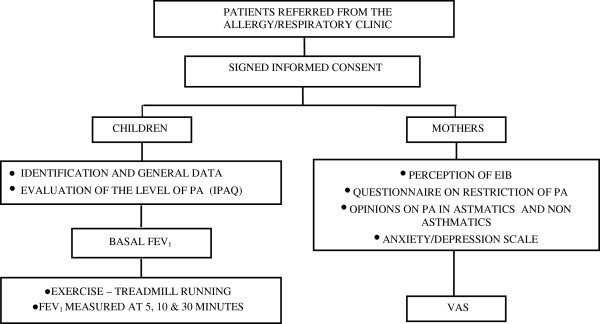
Study flow-chart.

Each stage was conducted by trained researchers who were supervised by a coordinator and data were collected according to the flowchart shown in Figure 1. Participants unable to answer the questionnaire because they were illiterate were interviewed.

### Statistical analysis

After an initial analysis, mothers and their children were separated into two groups: one with mothers who reported imposing restrictions on their children’s physical activities and the other with mothers who did not. Each of the predictive variables was checked by comparing proportions using the Chi-squared test and odds ratio (ORs) with 95% confidence intervals (CIs) in order to select those that were associated with mother’s attitude towards PA. These variables were then used for the construction of a multivariate regression model. The visual analog scale was dichotomously categorized as < 5 cm and ≥ 5 cm. HADS anxiety and depression scales scores were categorized as > 9 and ≤ 9 as this cut off point showed the best sensitivity/specificity discrimination relationship [25]. To check the association between the mother’s attitude and the children’s PA level, the chi-square test was used. A probability < 0.05 was considered significant. Data were analyzed using SPSS for Windows, version 12.0 (SPSS Inc, Cary, North Carolina- USA).

## Results

Evaluations were made on 115 pairs of mothers and their offspring. The children’s general characteristics are shown in Table 1. Of the 115 children/adolescents, 8% were obese (BMI > 30), 7% were overweight (25 < BMI < 29.9), 82% had normal weight (18.5 < BMI < 24.9) and 3% were underweight (BMI < 18.5). Of the 115 mothers, 10% were illiterate, 63% had completed elementary school and 27% high school.

**Table 1:** General characteristics of the asthmatic children and adolescents

	**Offspring**
**Total (n)**	115
**Age - years (average ± sd**^**‡**^**)**	13,06 ± 2.12
**Gender (M/F)**	67/48
**Weight - Kg (average ± sd)**	47.5 ± 11.8
**Height- cm (average ± sd)**	155.7 ± 9.2
**BMI – Kg/cm**^**2 **^**(average ± sd)**	19.3 ± 3.2
**Severity of the asthma – n (%)**	
Intermittent and mild persistent	70 (61%)
Moderate and severe	45 (39%)
**Medication - n (%)**	
No medication	7 (6%)
Only beta^2^-bronchodilators for relief	77 (67%)
Preventive treatment*	31 (27%)
**Monthly family income**^**† **^**- n (%)**	
≤ 1 minimum wage	54 (47%)
> 1 < 2 minimum wage	42 (37%)
≥ 2 minimum wage	19 (16%)

*Inhaled steroids, whether or not associated with long-acting beta^2^ adrenergic bronchodilators. ^**†**^Minimum monthly wage at the time of the survey = 280 U$ dollars. ^‡^Standard deviation.

Of all the mothers, 37% said they impose restrictions on their children’s physical activities (sports/games) in periods when they were not suffering an asthma exacerbation, and 63% said they did not limit PA. Table 2 shows the results of univariate analysis of associated factors. The mother’s restrictive attitude was not associated with the physical activity levels of their children (p = 0.9) nor BMI index, gender, medication use or family income (p = >0.99, p = 0.70, p = 0.82, p = 0.47 respectively). Also, there was no association between children’s asthma severity and PA levels (p = 0.58).

**Table 2:** Factors associated with the mother’s attitude towards physical activities – univariate analysis – (n = 115)

	**Mother’s attitude**			
	**Restricts**	**Does not restrict**	**OR (95% CI)**	**p**
	**n**	**n**		
	**43 (37%)**	**72 (63%)**		
**Does your child wheeze and/or have asthma when at play or taking part in sports?**				
Yes	35 (81%)	41 (57)	3.3	0.01
			1.3-8.1	
VAS* score after exercise				
< 5 cm	22 (51%)	53 (74%)	0.4	0.02
≥ 5 cm	21(49%)	19(26%)	0.2-0.8	
**Is doing exercise important for children?**				
Yes	42 (98%)	69 (96%)	1.8	>0.99
			0.2-18.1	
**Are you afraid that your child will fall ill if he/she does exercise?**				
Yes	32 (74%)	24 (33%)	5.8	< 0.001
			2.5–13.7	
**Is physical exercise dangerous for children with asthma?**				
Yes	23 (53%)	19(26%)	2.6	0.02
			1.2–5.8	
**Anxiety: HADS**^**¥**^				
> 9	25 (58%)	26 (36%)	2.5	0.03
≤ 9	18 (42%)	46 (68%)	1.1–5.3	
**Depression – HADS**^**¥**^				
> 9	13 (30%)	13 (18%)	2.0	0.2
≤ 9	30 (70%)	59 (82%)	0.8-4.8	
**Asthma severity**				
Intermittent and mild persistent	19 (44%)	51 (71%)	0.3	0.008
Moderate and severe	24 (56%)	21 (29%)	0.1–0.7	
**EIB**^**£**^				
Yes	25 (58%)	27 (38%)	2.3	0.05
			1.1–5.0	

*VAS – visual analog scale; ^¥^HADS – hospital anxiety and depression scale; ^£^EIB – exercise-induced bronchospasm.

After the multivariate analysis, it was found that the fear that the child would fall ill if he/she does exercises was significantly associated with the mother’s PA restriction. Other factors that were significantly associated with the mother’s PA restriction were the mother’s perception of dyspnea after the children running on the treadmill (VAS), mother’s anxiety level and children’s asthma severity (Table 3).

**Table 3:** Factors associated with the mother’s physical activities restriction – multivariate analysis*

**Factors**	**OR**	**95% CI**
**Are you afraid that your child will fall ill if he/she does exercise?**	7.96	2.97–21.29
**Dyspnea perception 5 min after exercise – VAS score**	3.27	1.20–8.92
**Anxiety – HADS**	3.36	1.30–8.70
**Asthma severity**	2.59	1.03–6.50

*Logistic regression; VAS – visual analog scale; HADS – hospital anxiety and depression scale; OR – odds ratio; 95% CI – ninety five percent confidence interval.

## Discussion

Our results showed that more than one third of the mothers of asthmatic children and adolescents admitted imposing restrictions on their children’s PA. This is probably a source of important conflicts of the mothers with themselves and with their children and may contribute to some of the children being less active. The factor that was most strongly associated with the mother’s attitude to imposing restriction was the fear that their children would fall ill if they do exercise, followed, in decreasing order, by maternal perception of dyspnea presented by their children after running on a treadmill, the level of the mother’s anxiety and asthma severity.

To find that almost all mothers recognize exercise as being important for children is encouraging. However, half of them admit fearing that their children will get sick if they do exercise and over one third believe that exercise is dangerous for children with asthma. These negative conceptions are in agreement with the findings of Lang et al. [15] in an urban population in the Northeast of the United States. They also found that these conceptions, associated with the idea that the children felt uncomfortable after exercising, were responsible for asthmatic children’s lower PA levels. Mansour et al. [29] also found that parents who were misinformed and regarded asthma as a barrier to doing PA unnecessarily restricted their children’s participation in sports and games.

Parent’s conceptions and attitudes towards sports and other types of PA may influence children’s own perception of their ability to participate [11,12] and those who attribute risks to children and adolescents with chronic diseases taking part in sports, games or playing around may contribute to their children having lower self-esteem and discourage them from doing PA [14]. On the other hand, Feredey et al. [13] observed that when parents had positive attitudes and opinions regarding doing physical activity, their children were more involved and committed to sports.

We cannot overlook the fact that a larger percentage of mothers than the one we found may be preventing their children from doing PA, given the number of mothers who claimed not to restrict their children’s PA but responded affirmatively to the questions “Is exercise dangerous for children with asthma?” and “Are you afraid that your child may fall if he/she does exercise?”. Mansour et al. [29] and Williams et al. [12] reported that even parents who say they do not limit their children’s physical activity are distressed by the possibility of children’s worsening of asthma symptoms which suggests that they may be, consciously or unconsciously, restricting or, at least, not encouraging their children to do such activities.

Mothers may also be preventing their children from doing PA based on the symptoms they perceive when their children are playing or taking part in sports. Firrincieli et al. [30], investigating physical activity in 3 to 5 year-old children, found that those with a history of asthma or wheezing in the last 12 months or that had been to an emergency room because of asthma were less active and suggest that this could be related to parents restricting the physical activities of their children.

The univariate analysis showed an association between the restrictive attitude of mothers and the occurrence of EIB but this association did not remain significant in the multivariate analysis. The perception of symptoms of dyspnea associated with physical activity both by mothers and by the patients themselves is flawed and is, very often, more related to a lack of being physically fit or to clinical states other than EIB. The diagnosis of EIB based solely on the perception of the symptoms should be taken with caution in the absence of any objective proof [7,31].

Our findings also showed that the mother’s level of anxiety was associated with their restrictive attitude to their children doing PA. No articles in the literature were found that have investigated this association. No association of the mother’s level of anxiety was found between the severity of the asthma or with the perception of dyspnea measured by the visual analogue scale. The presence of higher levels of maternal depression was another hypothetical factor that we considered as being able to influence the mother’s attitude, but no significant association was found.

The severity of asthma was associated with mothers’ restriction on physical activity in the multivariate analysis but, in spite of this, no significant association was found between the severity of asthma and the children’s PA levels. Mancuso et al. [32] reported an association of lower PA levels and asthma severity but in their research the patients themselves expressed the belief that exercise was not good for asthma.

Despite the importance of physical activity for children and adolescents with asthma, more than one third (35%) of the patients evaluated were considered inactive. This proportion did not differ from what has been found among students from 9th grade (12–16 years old) in the city of Recife who were evaluated in a large population-based epidemiological study conducted in Brazil (National Survey of the Health of Schoolchildren) [33]. Only a small proportion (14%) of our children and adolescents sample was engaged regularly in sports. In the remaining, PA was restricted to walking or cycling between home and school, occasional participation in football matches (soccer), games and other leisure activities.

Although a considerable number of the children in the study were classified as inactive, mothers restricting PA was not the determining factor for this. It is likely that despite mothers advising their children to avoid PA, they do not follow this advice and take part in PA when not under supervision, such as, in school. Some authors have shown that mothers have little knowledge of their children’s PA [10,12,18]. Interestingly, in a previous study we found an association between children’s reports of mother’s restrictive attitudes and a lower level of PA, suggesting that when the mother’s prohibitions are taken into account by their children this can have an impact on their level of physical activities [34].

A possible bias in our results is the fact that the instrument used to assess the PA level in children and adolescents – IPAQ – does not provide precise and direct measurements of energy expenditure and questionnaires typically require respondents to recall their activities over the determined period [24]. Although assessing the PA level by using questionnaires for children and adolescents is still an issue of debate in the literature [35,36], we used the short version of the International Physical Activity Questionnaire (IPAQ) that is validated and formally translated to Portuguese (Brazil) has acceptable measurement properties on data obtained on participants from 12 countries, including Brazil [24]. Another possible bias is that it was not included in the questionnaire a query for mothers about maternal asthma. Mothers that have experienced the feeling of dyspnea could impose more physical activity restriction to their children.

## Conclusions

Many mothers of asthmatics children and adolescents believe that PA can worsen their children’s asthma symptoms and this is the most important factor associated with their restrictive attitudes towards physical activities. As this behavior may discourage children from having an active healthy lifestyle, our findings reinforce the need for physicians and health professionals to address and discuss these misconceptions with patients and their caregivers. With appropriate evaluation, effective preventive treatment and counseling these children do not need to avoid physical activities and can be stimulated to have a fully active life.

## Competing interests

The authors declare that they have no competing interests.

## Authors’ contributions

FMNAD Contributed to data collection and analysis. MAVC Contributed to data collection and analysis. ARS Contributed to Sudy design and data analysis. DMP Contributed to Study design and data collection. ESCS Contributed to Data analysis and manuscript writing. JAR Contributed to Study conception, data analysis and interpretation and manuscript writing. All authors read and approved the final manuscript.

## Pre-publication history

The pre-publication history for this paper can be accessed here:

http://www.biomedcentral.com/1471-2458/14/287/prepub
